# The Role of Neighbours Selection on Cohesion and Order of Swarms

**DOI:** 10.1371/journal.pone.0094221

**Published:** 2014-05-08

**Authors:** Angelo M. Calvão, Edgardo Brigatti

**Affiliations:** 1 Instituto de Física, Universidade Federal Fluminense, Campus da Praia Vermelha, Niterói, RJ, Brazil; 2 Instituto de Física, Universidade Federal do Rio de Janeiro, Cidade Universitria, Rio de Janeiro, RJ, Brazil; University of Zurich, Switzerland

## Abstract

We introduce a multi-agent model for exploring how selection of neighbours determines some aspects of order and cohesion in swarms. The model algorithm states that every agents' motion seeks for an optimal distance from the nearest topological neighbour encompassed in a limited attention field. Despite the great simplicity of the implementation, varying the amplitude of the attention landscape, swarms pass from cohesive and regular structures towards fragmented and irregular configurations. Interestingly, this movement rule is an ideal candidate for implementing the selfish herd hypothesis which explains aggregation of alarmed group of social animals.

## Introduction

Collective group motion is an important biological phenomenon that has received much empirical and theoretical attention from investigators in disciplines as varied as computer science [1], biology [2]–[6] and physics [7]–[9]. Generally speaking two types of mechanisms are considered: an aligning interaction, and attraction/repulsion between individuals [10]. Usually, the first one is the responsible for the emergence of polarised groups [11], the second one for maintaining cohesion with a rather homogeneous density which can correspond to a certain level of regularity in the spacial distribution. In this work we will focus only on the second.

Cohesion and regularity in the spacial distribution can generate many biological advantages.

First, we can list energetic benefits. A classical example is given by flocks of birds which align themselves in “V” formations [12]. In this situation, individuals seek an optimal mutual position which generates a regular structure in the distribution of inter-individual distances. Other examples of this behaviour can be found in the core of big herds of migrating mammals [4], and in flocks of surf scoters moving on the water surface. Very detailed observations showed that individuals seek a target density searching for an ideal distance from the other components of the group, generating well-defined spatial structures [13]. As inferred by a recent field study on mosquitofish [14], on the one hand, animals move away from individuals that intrude their personal space, defining a stress zone, while, on the other hand, they are attracted to individuals at a significant distance. This behaviour allows to maintain integrity as a group while decreasing the frequency of abrupt accelerations or decelerations.

Second, aggregation can improve reproductive success [15]. An astonishing case is displayed by the males of a cichlid fish which mark their breeding territories digging pits. They design hexagonally shaped territories that reach an impressive high degree of order [16].

Finally, a particularly important benefit is the reduction of predation risk. The study of this aspect generated an interesting conjecture for explaining aggregation of individuals [17]. This idea, known as the “selfish herd hypothesis”, suggests that cohesive swarms are generated because individuals move toward one another for minimising their own predation risk. Some recent field observations presented evidences of this behaviour analysing the movements of sand fiddler crabs [18] and seals [19]. This last study revealed that simple movement rules are used to reduce predation risk. Effectively, a successful implementation of this hypothesis require an individual movement rule as simple as possible. This is a necessary condition for many species, across different taxa, being able to follow it and for enabling natural selection to fix it [20].

Unfortunately, simple rules tested by computer simulations seems to fail to produce aggregation [21], [22]. Specifically, the implementation of the Hamilton's algorithm, where the focal individual moves towards the nearest neighbour, does not result in compact and dense groups and fragmentation into multiple sub-groups of a few individuals occurs. Discovery an elementary movement rule which can produce compact aggregation is still an unsolved problem which has been denominated the “dilemma of the selfish herd” [22].

In the following we introduce a very simple algorithm which seems to find a solution to this dilemma: we consider an interaction where individuals move toward the nearest neighbour encompassed in a limited attention field. We stress how this rule is more natural than asking individuals to make their decisions averaging over the influence of many neighbours [3], [8], [22]. In fact, in our case, cohesion is determined by the number of interactions and not by abstract parameters which control the interaction like in a molecular type force. It is difficult to imagine that organisms behave like particles in a physical system, where interaction is mediated by a potential which directly sums up for all the neighbours. Individuals must undertake efficient decision-making, instead of relying on the weighted sum of a large number of neighbours. For this reason, they choose only the neighbours relevant for their purpose and they face a natural limitation over the information they can handle. These limits are not just shaped by the physiology of vision or the visual system response; perceptual and cognitive effects should be the most relevant ones. Among them, the physiology of attention should be particularly important. When we are confronted with a large number of items, we withdraw from most and we focus our attention on just a few. The visual system's neurones are responsive to what neighbours we are interested in [23]–[25]. We simplify these considerations imagining that individuals make decisions based on an angular landscape defined by their attention. In this attention field, the organism fixes on the closest neighbour and reaches a preferred distance in relation to it. The fact that this rule is based on a topological interaction and depends on perceptual limits are realistic aspects outlined by field observations [26], [27]. Moreover, a recent study, which explicitly determined the interaction rules in fish groups, identified the single nearest neighbour interaction, applied with the aim of active regulating the distance between pair of animals, as the principal mechanism for collective motion [14].

Our simple algorithm is capable of exhibiting a rich behaviour characterised by different phases. Changing the amplitude of the attention landscape swarms pass from cohesive and regular structures towards fragmented and irregular configurations. Different levels of angular and positional order are spanned and described. In particular, it becomes evident that only dealing with a reduced portion of the attention field can generate a cohesive and ordered swarm. In this regime the algorithm is an ideal candidate for implementing the selfish herd hypothesis.

The paper is organised as follows. In the following section we introduce the model. In Section *Analysis of the model behaviour*, an in depth analysis of the algorithm is performed, giving a clear overview of its performance in different regimes. In Section *Application to the selfish herd problem*, we apply our movement rule to the selfish herd problem and we compare qualitatively our results with field observations. Conclusions are reported in the last section.

### The Model

We consider a system composed by 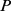 agents which move continuously on a square of linear size L and which stop if they reach the boundary. In any case, the definition of the specific form of the boundary condition is not relevant, because, in practice, individuals never approach the boundary. The time unit 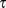 is the time interval between two updatings of the positions of all the agents. In most of our simulations the initial conditions correspond to 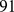 individuals uniformly and randomly distributed on the plane with a density 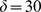. The stress zone radius 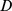 is fixed to 0.1 length unit.

Movements are determined asynchronously. An agent 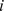, with position 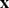, is randomly selected and a gazing direction is assigned by a random number chosen with a uniform probability from the interval 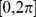. This probability distribution is the simplest hypothesis for a general implementation of our algorithm and it can be supported by some observational results [13]. The gazing direction is the bisector of the angle 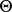, which defines the attention field where the nearest neighbour is sought. It is important to implement this search by using a fast algorithm. We employed the Computational Geometry Algorithms Library, using the 2D Range and Neighbor Search [28].

Given the position 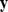 of this neighbour, if 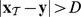, 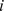 moves in the direction of its neighbour a step 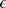: 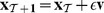, where 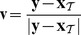. If this movement results in the stress zone invasion, 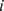 stops along the direction 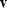 at a distance 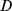 from its neighbour.

**Figure 1:**
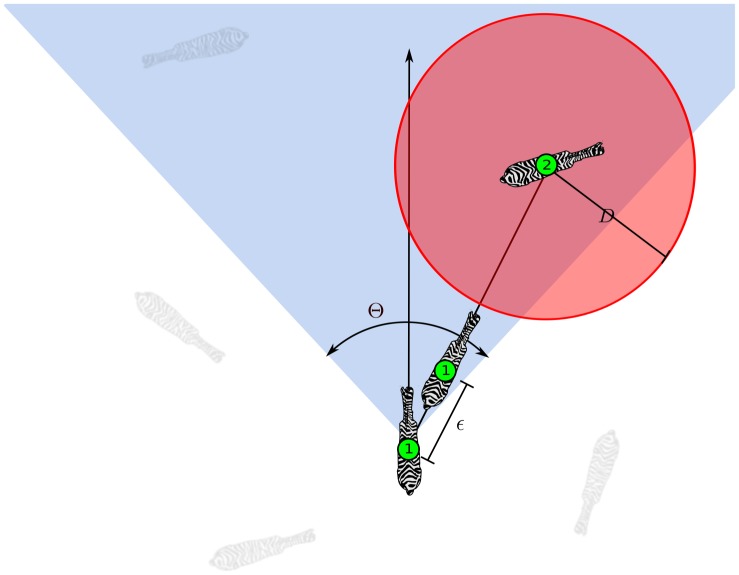
An example of an agent's movement. Individual 1, moves with speed 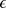 towards individual 2 which is the nearest neighbour encompassed by the attention field, the blue region characterised by the angle 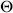. The stress zone of individual 2 is the red disk of radius 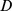.

If 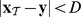, the movement is: 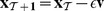. If 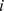 gets farther than 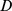 from its neighbour, 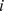 stops at a distance equal to 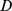. Figure 1 gives an example of these simple rules.

This algorithm considers only the relative movements of the individuals. Obviously, for describing a directed moving swarm, a superimposed common collective velocity can be added but it is not relevant for our analysis.

Simulation parameters are summarised in Table 1. Our C++ computer code is available upon request.

**Table 1:** Parameters of the model.

Parameter	Description	Typical value	Biological interpretation	Typical unit
D	stress zone radius	0.1	average inter-individual distance	10 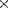 m
L	linear size of the box	1.74	linear size of the observation region	10 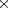 m
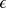	speed	0.0112	average velocity	10 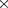 m/s
P	group size	91	group size	individuals
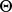	amplitude of the attention field	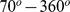	average attention field	degree

The typical values are used in the simulation trials of section *Analysis of the model behaviour* and, in this case, 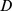, 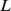 and 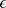 are expressed in length units. The column “typical unit” refers to the simulation of a swarm of crabs [18] as described in section *Application to the selfish herd problem.*

## Results

### Analysis of the model behaviour

The principal purpose of this section is to quantify, with respect to the amplitude of the attention field, which degrees of cohesion and order our algorithm is able to produce. For this reason, the algorithm operates until a quasi-stationary state is reached. We consider this state because a neat and clear analysis can be performed and not because we are interested in static aggregation. In fact, the active configurations displayed along the dynamics are qualitatively identical to the final quasi-stationary state. We affirm that our system entered this state if, during 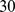 time steps, no changes in agents' positions is recorded. This state can correspond to an effective absorbing state, where all the agents' distances with all their topological neighbours are equal to 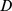. Otherwise, it is possible that some movements, even if improbable, would be still feasible. In this last case, the agents' distances from their first metric neighbour are equal to 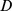.

**Figure 2:**
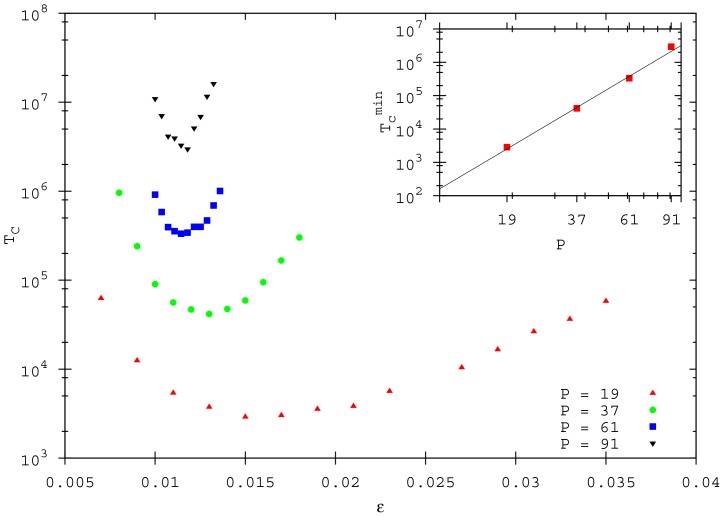
The convergence time as a function of the speed 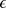, for different populations 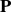 (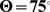, 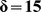). Data points represent averages taken over 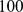 different simulations, where the individual initial distribution is different. In the inset: 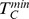 as a function of 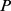. The continuous line is the fitted relation: 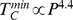.

First, we investigate the behaviour of the convergence time for reaching a quasi-stationary state (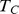). Interestingly, the 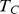 value strongly depends on 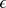 and an optimal 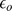 value exists, for which 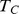 is minimal (Figure 2). In addition, varying the population size, the optimal 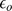 value slightly changes along with the value of 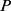. Since we start all simulations with the same density, different convergence times are not caused by different density values, but by a collective effect in the ordering procedure. In Figure 2 we display the convergence times for different population size when the 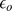 value is chosen (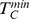). As can be appreciate, 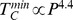.

Now we focus on the description of the dynamics of the model. We introduce two order parameters which are able to capture the degree of order reached by the swarm. We characterise the degree of positional orientational order of a given configuration defining, for each organism 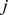 [29], [30]: 
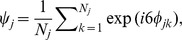
 where 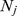 is the number of topological neighbours of individual 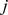, which are the organisms whose Voronoi polygons share an edge with individual 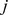. Finally, 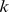 is the index of the neighbours and 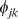 is the angle relative to the bond between 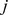 and 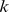 and an arbitrary fixed reference axis. The factor of 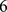 is introduced for detecting perfect sixfold ordered structures. A positional orientational order parameter is given by the norm of the average of 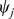 over all the organisms 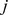:
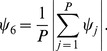
(1)


**Figure 3:**
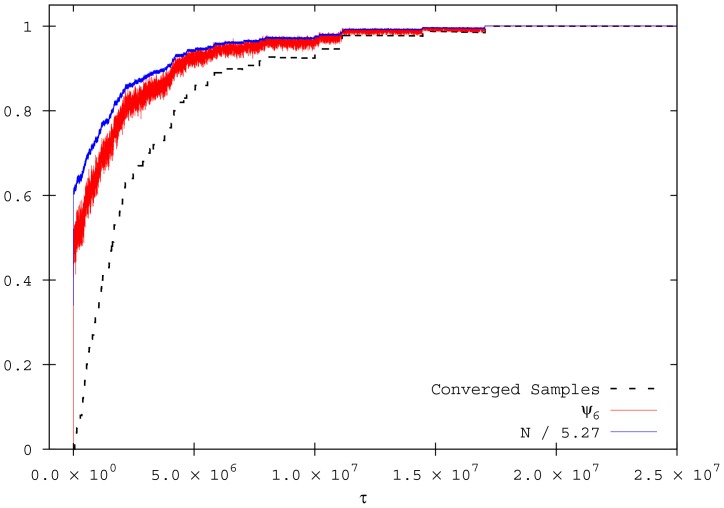
Time evolution of the order parameters 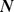 and 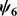 averaged over 100 simulations. The dashed line is the convergence probability of the ensemble of simulations. 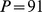, 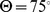, 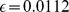, and 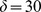.

Translational order can be investigated by looking at the sum of the number of individuals contained in a circle of radius 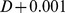 around a given individual. We obtain the translational ordering parameter 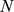 averaging this quantity over all the individuals. Figure 3 shows the time evolution of these two order parameters for typical values which generate a final state characterised by a perfect sixfold ordered structure (absorbing state).

**Figure 4:**
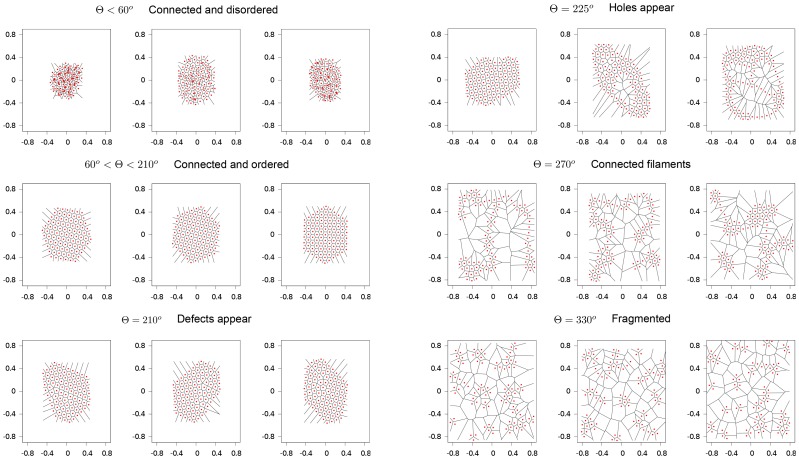
Examples of some characteristic configurations of the quasi-stationary states for different 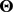 values (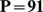, 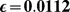 and 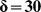). The red dots represent the agents' position and the lines depict the Voronoi tessellation.

In the following we analyse the cohesion and order of the swarm varying the attention field angle 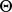. For this purpose, we look at the quasi-stationary states, which clearly reveal the differences in the ordering ability of the algorithm for different values of 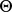 (see Figure 4). This fact forces us to run very long simulations where almost the entire computational time is lost in searching for the nearest neighbour inside a given attention field.

For small 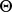 values the swarms maintain a high level of cohesion, but a totally disordered configuration. The groups reach a high density and individuals do not respect the stress zone. The quasi-stationary states are not attained and individuals continue to change their relative positions. For 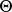 values higher than 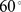, the interaction arranges the swarm in the densest way compatible with a pairwise distance equal to 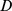. In fact, all the organisms are located on the vertices of equilateral triangles, which tile the plane along the six-fold symmetric triangular lattice. This phase can be easily detected looking at the value of 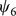 which is equal to 1. Raising the value of 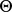 around 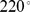 lattice defects begin to appear in the form of individuals with a number of neighbours different from 6 (generally 5 and 7). The disordered phase (liquid) emerge for 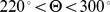. In this interval, increasing the value of 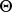 generates holes in the swarm structure. These ruptures can significantly grow generating linear structures of particles, which result in sub-swarms connected by filaments. Finally, for larger 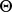 values (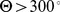) cohesion is lost and isolated clusters of organisms appear.

**Figure 5:**
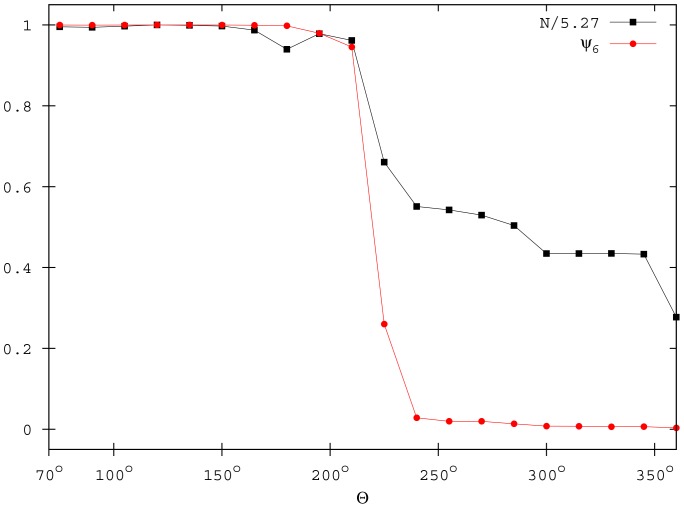
Order parameters 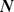 and 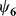 for different 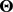. Data are averaged over 100 simulations when the system reaches the quasi-stationary state (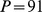, 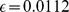, and 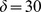).

**Figure 6:**
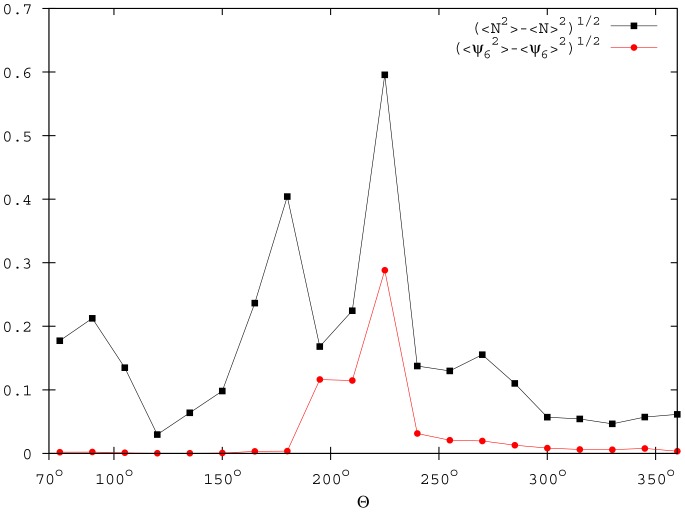
The standard deviations of 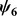 and 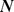 for different 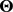. Data are averaged over 100 simulations when the system reaches the quasi-stationary state (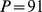, 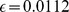, and 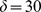).

An abrupt variation in the 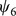 and 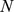 values signal these transitions (Figure 5). The transition between the ordered and the disordered phase (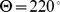) is clearly detected by the drops in the 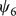 and 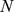 values. Moreover, in analogy with equilibrium phase transitions, the fluctuations of the order parameters increase on approaching this critical value. This is shown in Figure 6 by the standard deviations of 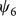 and 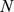, which exhibit a sharp peak in correspondence of 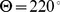. The second transition between the cohesive and the fragmented phase (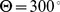) is evidenced by a decrease in the 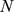 parameter, which reaches a plateau value close to 2.

To sum up, we can highlight four different phases: a first disordered, highly dense, connected phase (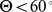), a crystal-like phase, with hexagonal patterns (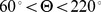), a low ordered phase with the presence of holes and ruptures and, finally, for 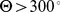, a fragmented swarm, where the initial group splits in different clusters and cohesion is lost. An interesting dependence of this behaviour on the value of the initial density was found and will be published any time soon.

A natural interest exists for introducing a noise source in our ordering algorithm. Noise is an obvious element presents in real systems and configurations obtained with the presence of noise can have a stronger relation with real-life situations. In addition, from a theoretical point of view, it is interesting to state if this new ingredient can generate some type of order-disorder phase transition. For this reasons, we introduce a noise in the evaluation of the direction of the displacement of each organism. With this new rule, an agent, after having determined the vector 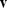, changes the orientation of its movement by a random angle chosen from the interval 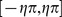 with a uniform probability. This means that the final direction of the movement is obtained after rotating the original direction 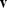 with a random angle and 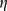 is the parameter which controls the noise strength. The speed continues to be equal to 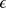, with the same restriction of the deterministic case when the ideal distance 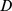 is crossed.

**Figure 7:**
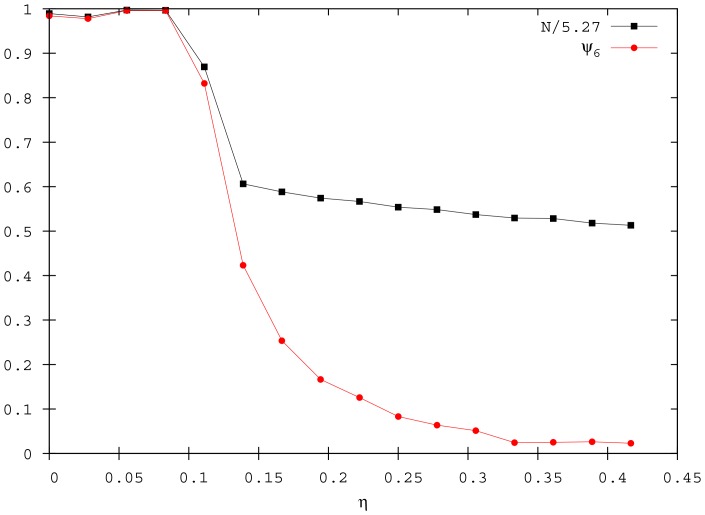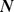 and 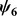 as a function of the noise strength 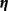 after 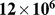 iterations. Data are averaged over 100 simulations (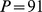, 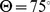, 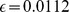 and 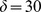).

We fix 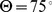, which, for 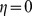, generates the ordered six-fold configurations. As can be seen in Figure 7 an order-disorder transition emerges, where the disordered phase is characterised by 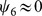. We can clearly appreciate the abrupt appearance of a spatial order for a critical value of the noise, where a collective motion is attained for sufficiently low levels of noise.

### Application to the selfish herd problem

In the following, we connect the results of our analysis with the problem of finding a good movement rule for the selfish herd hypothesis. To satisfy this hypothesis our algorithm must be able to generate a single densely packed cluster of individuals with a minimal area of the Voronoi polygons (small domains of danger) [22]. Considering that we model a system with individuals characterised by a stress zone which is controlled by the parameter 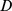, the ideal packed aggregation corresponds to a regular triangular lattice of side 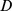. It is clear that for 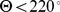 our algorithm achieves a perfect solution for this problem. In contrast, if we chose 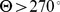 we generally obtain the same unsatisfactory results described in references [21], [22], which are characterised by a lost of cohesion. In particular, simulations with 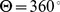 correspond to the original Hamilton's rule [17]. To reinforce these considerations we calculate, for the same parameters of Figure 5, the mean area of the polygons at time 0 and for the final configuration. Averaging over different simulations, we find that for 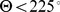 there is a reduction of the polygons area, and thus, of the domain of danger, of up to 75%. Increasing the 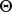 value this reduction diminishes and it disappears for 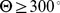.

**Figure 8:**
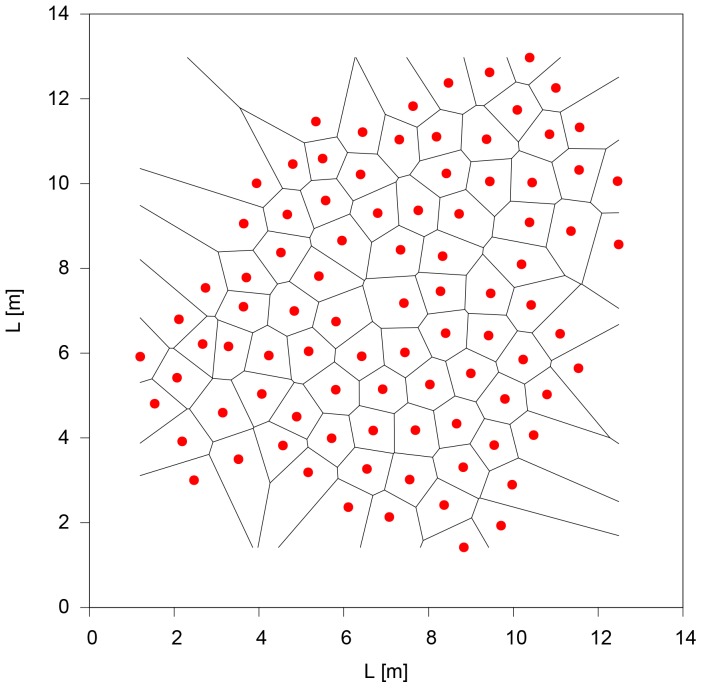
An active configuration obtained using the parameter 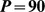, 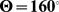, 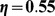, 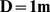, 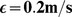, and 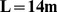. After a rapid transient, during which the group density increases, we obtain a configuration qualitatively similar to the field observations of reference [18].

Finally, we qualitatively compare the outputs of our algorithm with the field observations of crabs groups [18]. We consider simulations where noise is in action and before they reach a possible absorbing state. As far as data are available, we parameterise our model to realistic values. The parameter 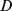 corresponds to the mean inter-individual distance after the attack (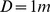), the speed to the average crabs velocity during the attack (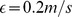), and 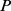 is fixed to 90 individuals. For a square of linear size 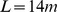 the corresponding initial density can be put in relation with the observed one. If the attention field 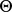 is tuned to values smaller than 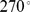, for a wide range of 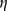 values, after a rapid transient we can observe lively shrunk configurations qualitatively very similar to the observed real data (see Figure 8).

**Figure 9:**
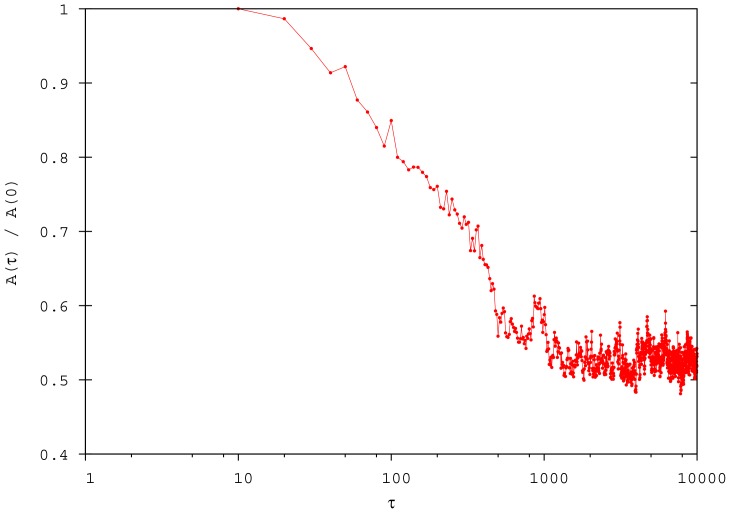
Time evolution of the sum of the areas of the Voronoi polygons (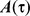), normalised for its value at 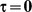. A significant reduction of the domain of danger is obtained after a few iterations of the algorithm.

For this set of parameters, we can state how realistic our simulations behave. If we consider that fixing 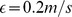 implies that our time unit 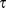 corresponds to a second, after a few seconds a significant reduction of the domain of danger is obtained, and after a few minutes the ideal compact configuration is reached (see Figure 9). These results can be realistically compared with the experimental facts.

## Discussion

We introduced a simple model for exploring some aspects of order and cohesion in swarms. The model consists in a straightforward algorithm which states that every agents' motion seeks for an ideal distance from the nearest topological neighbour contained in a given attention field. This approach is based on two fundamental facts present in nature: the existence of a limited attention field and the necessity for a decision-making based on a minimal rule. Despite the great simplicity of the implementation, varying the amplitude of the attention landscape, the model generates a very rich behaviour: swarm can maintain a disordered connected shape, it can crystallise in a six-fold ordered lattice, it can display a low ordered phase or it can fragment and lose cohesion. Moreover, introducing a source of noise, an order-disorder transition naturally appears. These results are significant for several reasons.

First, this interaction is an ideal candidate for solving the “dilemma of the selfish herd” [22]: to be able to find an easy movement rule that can produce dense aggregations. Until now computer simulations have failed to obtain a large compact aggregation generated by the algorithm proposed by Hamilton: approaching the nearest neighbour does not result in a large, dense group. In contrast, the simple introduction of a reduced attention field is able to produce the densest aggregation in a centrally compact swarm, with a reduction of the domain of danger [17], when some values of the angle which defines the attention field are selected. In this perspective, the presence of a limited attention field can be interpreted not only as a consequence of the constraints in the information access or in cognitive abilities, but as an active regulation for reaching a specific collective spatial configuration. Animals may use attention mechanisms to switch between processing few stimuli or many [31] in dependence of their objectives. In our model these adjustments are obtained modifying the value of the parameter 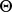. A single animal can switch from high values of 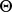, in situations of low predation risk and foraging, which correspond to sparse and disconnected configurations, to small values, when facing situations of danger, which correspond to compact and dense configurations.

Second, this dynamical rule, capable of generating spatial structures with specific geometrical constraints, has a general interest for collective aggregation. In fact, these spatial structures can be related to the distribution of mutual distances observed in surf scoters which form well-spaced groups of individuals on the water surface [13]. Moreover, our results can give some insights for other conventional models of collective motion. As our study clearly evidences, local interactions with few topological neighbours are not just economic, but the more efficient way of granting the highest levels of cohesion and coherence in the swarm. In contrast, the interaction with a larger number of individuals results in a loss of order and cohesion. These outcomes are supported by experimental evidences in mosquito-fishes [14], where the active regulation of the distance to the single nearest neighbour is the fundamental interaction rule, and they are in line with the results of different models which show that smaller view angles allow better cohesion and a faster dynamics towards polarisation [32], [33].

Third, our results could be transposed into practical applications for designing artificial swarms builded up by cooperative mobile robotics [34], [35]. The studied empirically-based algorithm could be encoded into instructions for a scheme of distributed coordination to guarantee collision avoidance and cohesiveness in groups of autonomous agents. From our results it follows that, for some values of the parameters, the final swarm configuration maximises the coverage of a given environment, a fact that can be useful for communication or detective purposes [36]. A specific target application could be a swarm of mobile robots for environmental monitoring.
